# An Organismal Model for Gene Regulatory Networks in the Gut-Associated Immune Response

**DOI:** 10.3389/fimmu.2017.01297

**Published:** 2017-10-23

**Authors:** Katherine M. Buckley, Jonathan P. Rast

**Affiliations:** ^1^Department of Biological Sciences, The George Washington University, Washington, DC, United States; ^2^Department of Pathology and Laboratory Medicine, Emory University School of Medicine, Atlanta, GA, United States; ^3^Department of Medical Biophysics, University of Toronto, Toronto, ON, Canada; ^4^Department of Immunology, University of Toronto, Toronto, ON, Canada

**Keywords:** inflammation, pigment cells, interleukin 17, gut immunology, phagocytosis, echinodermata, larva, sea urchins

## Abstract

The gut epithelium is an ancient site of complex communication between the animal immune system and the microbial world. While elements of self-non-self receptors and effector mechanisms differ greatly among animal phyla, some aspects of recognition, regulation, and response are broadly conserved. A gene regulatory network (GRN) approach provides a means to investigate the nature of this conservation and divergence even as more peripheral functional details remain incompletely understood. The sea urchin embryo is an unparalleled experimental model for detangling the GRNs that govern embryonic development. By applying this theoretical framework to the free swimming, feeding larval stage of the purple sea urchin, it is possible to delineate the conserved regulatory circuitry that regulates the gut-associated immune response. This model provides a morphologically simple system in which to efficiently unravel regulatory connections that are phylogenetically relevant to immunity in vertebrates. Here, we review the organism-wide cellular and transcriptional immune response of the sea urchin larva. A large set of transcription factors and signal systems, including epithelial expression of interleukin 17 (IL17), are important mediators in the activation of the early gut-associated response. Many of these have homologs that are active in vertebrate immunity, while others are ancient in animals but absent in vertebrates or specific to echinoderms. This larval model provides a means to experimentally characterize immune function encoded in the sea urchin genome and the regulatory interconnections that control immune response and resolution across the tissues of the organism.

The enormous progress made in the recent years in the field of pathology will surely also fertilize the field of pure zoology and at the same time the evolutionary standpoint of the latter field can provide solutions to medical problems in a comparative pathologic way. [Elya Metchnikoff (1)]

## Conservation and Innovation in Animal Immunity

Immune systems mediate complex interactions between animal hosts and a community of microbes that includes both pathogenic and beneficial strains (2). These ongoing processes occur in cells and tissues that are located across the animal and must be regulated at an organism-wide scale. In this context, immune response can be described as a distributed network of interconnecting regulatory circuits that are coordinated to protect the host and stabilize interactions with microbiota. Given its central role in animal life, this integrated circuitry is, at some levels, subject to deep evolutionary conservation (3, 4). Consequently, causal connections gathered from experiments in morphologically simple invertebrate models have direct implications for understanding immunity in more complex vertebrates.

Most bilaterians harbor specialized immune cells that exhibit morphological or behavioral similarities (5, 6). One well-known example is the phylogenetically widespread phagocytic cells, which were first recognized and described in several invertebrates by Metchnikoff (7, 8). Dedicated phagocytes often exhibit similar motility and surveillance-like behaviors in different phyla. Many animal lineages also have granular cells that participate in immune sensing and control (5). Through intricately coordinated signaling mechanisms, these cell types cooperate to initiate and resolve immune response. In addition, immune cells express many rapidly evolving proteins such as non-self recognition receptors (9) and secreted effector molecules (10). Nonetheless, the characteristics of terminally differentiated immune cells (morphology and behavior) cannot be used to reliably infer evolutionary relationships among cell lineages. Instead, homology likely lies beyond cell lineages when comparing immunity in different phyla (i.e., the relevant unit of homology that is useful for understanding immune system evolution is likely to more often lie at the level of the regulatory subcircuitry within cells). Evolutionary pressure on immune systems manifests differently among gene types (11) but, in general, immune receptors and effectors tend to evolve quickly. and their relationships among phyla can be difficult to interpret. The regulatory circuitry that controls cell development and function can provide insight into this problem by defining the nature of homology in these systems across phyla.

## Echinoderm Larvae: A Not so Novel Model System in Immunology

Echinoderms, together with the hemichordates, form a sister group to Chordata at the base of the deuterostomes (12). This evolutionary distance [echinoderms and chordates diverged ~530 million years ago (13)] provides the opportunity to investigate varying scales of immune system evolution, including (1) common mechanisms that regulate immunity throughout the deuterostomes, (2) ancestral strategies present in invertebrate deuterostomes or throughout Bilateria but specifically lost in vertebrates, and (3) evolutionary innovations that are specific to echinoderms. Examples of all three are evident in the sea urchin larval immune system.

Most sea urchins have biphasic life histories that include relatively long-lived, morphologically simple, planktonic larval stages. This form of development is ancestral to echinoderms (14). In the purple sea urchin (*Strongylocentrotus purpuratus*), a single female produces millions of eggs that, once fertilized, synchronously develop over 5 days into a free swimming, pluteus larva that feed for about 2 months before metamorphosis into a benthic juvenile form [reviewed in Ref. (15)]. Larvae have a tripartite gut composed of an epithelial monolayer (16) and a cellular immune system of 80–150 mesenchymal cells that populate the blastocoel or are apposed to the ectodermal epithelia (17, 18). From an experimental standpoint, echinoderm larvae offer several advantages: transparency that enables organism-wide, *in vivo* imaging at single-cell resolution, and efficient transgenic strategies to precisely perturb protein function (19, 20). These characteristics can be exploited to investigate open questions in immunology.

## A Wealth of Echinoderm Genomic Resources is Available

Experimental studies in echinoderms are supported by an extensive collection of genomic resources [www.echinobase.org (21)]. The purple sea urchin was the subject of the first assembled genome from an outbred, motile marine invertebrate and the largest invertebrate genome (814 Mb) sequenced at the time (22). Analysis of the *S. purpuratus* genome sequence identified many features previously believed to be vertebrate specific that were instead deuterostome or bilaterian innovations. One of the most striking findings was the expansive repertoire of genes encoding proteins with roles in immune recognition and defense (22–24).

Specifically, *S. purpuratus* has orthologs of most major transcription factor subfamilies important in vertebrate immunity (23). These include factors that regulate gene expression in the course of immune response (e.g., NF-κB and IRF), as well as regulators of vertebrate hematopoiesis (25–27). Many homologs of vertebrate cytokines are absent, which is not surprising given the rapid evolution of these factors and their receptors even among vertebrates (28). However, the genome sequence contains homologs of tumor necrosis factor α, macrophage inhibitory factor and interleukin 17 (IL17), as well as IL1 receptors (23). This shared regulatory heritage between echinoderms and vertebrates enables experimental investigations into transcriptional control of immune cell development (25, 26) and immune response (17, 29) that can provide meaningful insight to vertebrate biology.

In contrast to this conservation, the *S. purpuratus* genome sequence contains surprisingly large families of genes that encode pattern recognition receptors. The repertoires of toll-like receptors (TLRs), NOD-like receptors, and proteins containing multiple scavenger receptor cysteine rich domains are significantly (~10-fold) larger than their counterparts in the well-characterized vertebrates and insects (23, 24, 30–32). The sea urchin TLRs form 10 subfamilies based on phylogenetic analysis (33). Genes within these subfamilies are differentially expressed in larval and adult tissues and are most highly expressed in the coelomocytes and gut tissue, which are both sites of dynamic immune activity. Residues predicted to be in close spatial proximity are subject to strong positive selection. The expression patterns, rapid evolution, and lack of expression during early development strongly suggest an immune role for the sea urchin TLRs (33). These and other immune innovations within the echinoderm lineage [e.g., the transformer (Trf, 185/333) proteins; reviewed in Ref. (34)] highlight the diversification of proteins that potentially interact directly with pathogens, as has been observed in other systems (11) and provide a rich platform to study the integration of these quickly evolving proteins with more conserved elements of regulatory circuitry.

The Sea Urchin Genome Project has also assembled genome sequences from two additional sea urchins, a sea star, sea cucumber, and brittle star (www.echinobase.org). Four high-quality and three less complete genome assemblies, as well as high coverage, unassembled whole genome sequencing reads are available from other echinoderm species (35–38). In total, the NCBI Short Read Archive hosts 206 projects in 75 echinoderm species that cover all five classes as of this writing. Collectively, these data provide deep coverage and broad evolutionary perspective for investigations of echinoderm immunity.

## Several Cell Types Mediate the Larval Immune Response

To understand how this genomic complexity is deployed *in vivo*, immune response has been investigated in sea urchin adults [reviewed in Ref. (39)], as well as the embryonic and larval stages (17). Early life stages offer significant experimental advantages for characterizing the gene regulatory networks (GRNs) that control immune cell development and immunity. The many experimental strategies designed to investigate developmental GRNs in sea urchin embryos (40) can be applied to investigations targeting immunity in the larva.

**Figure 1:**
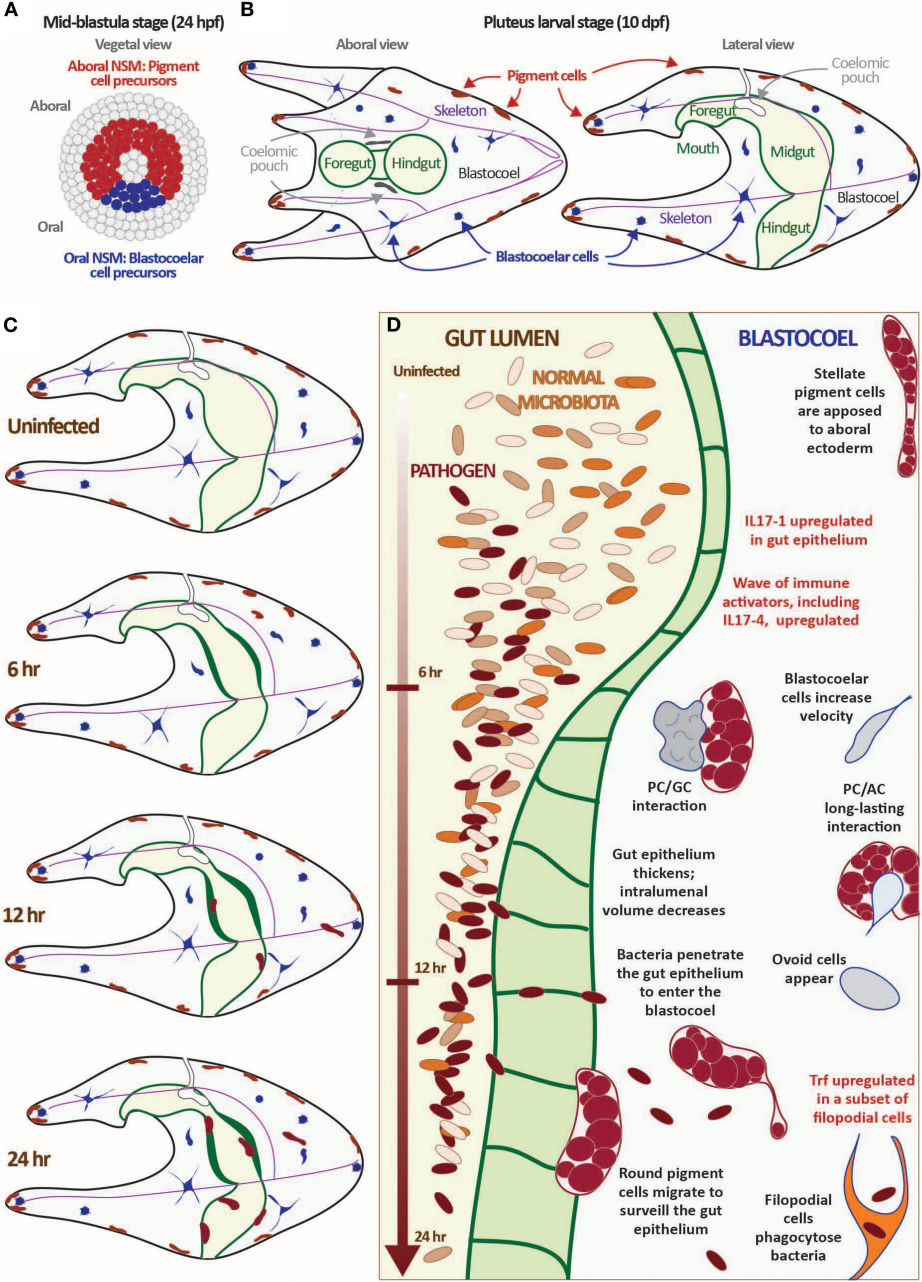
Exposure to the marine bacterium *Vibrio diazotrophicus* induces an acute gut-associated inflammatory response in sea urchin larvae. **(A,B)** Sea urchin larvae exhibit a cellular immune response mediated by several mesodermally derived cell types. The mesenchyme blastula-stage embryo is shown from the vegetal view **(A)**. In *Strongylocentrotus purpuratus*, embryos reach this stage about 24 hpf. The ring of non-skeletal mesoderm (NSM) cells is indicated by either red (aboral NSM) or blue (oral NSM). All other cell lineages are shown in gray. Aboral NSM cells differentiate into larval pigment cells; the oral NSM derivatives become the heterogeneous blastocoelar cells. Aboral and lateral views of the pluteus larvae are shown **(B)**. Morphological features are indicated (pigment cells, blastocoelar cells, celomic pouches, skeleton, and gut). The images shown in panels **(A,B)** are not to scale. **(C,D)** Larvae mount a cellular and transcriptional immune response to exposure to *V. diazotrophicus* in the sea water. In the first 24 h of exposure to *V. diazotrophicus*, the midgut epithelium thickens, reducing the volume of the gut lumen. Pigment cells change shape from a stellate to round morphology and migrate from the ectoderm to the gut. Cell motility increases, and complex cell:cell interactions occur. Bacteria begin to penetrate the gut epithelium and enter the epithelium, where they are phagocytosed by a subset of filopodial cells. One of the first transcriptional events is the acute upregulation of the *IL17-1* genes in the gut epithelium. This is followed by activation of a second wave of immune gene upregulation, including the *IL17-4* subtype. Immune effector genes, such as *Trf*, are activated in a subset of filopodial cells later in the response. Data are described in detail in Ref. (17, 29).

The larval immune response is mediated by a collection of phagocytic and granular immune cells [~100 total cells at 10 days post-fertilization (dpf) (17)]. These cells are initially specified in the early blastula-stage embryo from a ring of non-skeletal mesodermal (NSM) cells that differentiate into pigment cells, a heterogeneous suite of blastocoelar cells, and several other cell types including pharyngeal muscle and celomic pouches [Figures 1A,B; Ref. (25, 41)]. Presumptive pigment cells activate the transcription factor *glial cells missing* (*gcm*) and a battery of differentiation genes early in development that remain upregulated in the aboral NSM ring by late blastula (42). These cells migrate into the blastocoel relatively early in gastrulation and migrate to the aboral ectoderm and larval arms in an Ephrin/Eph receptor-mediated system (43). Differentiated granular pigment cells produce the antimicrobial naphthoquinone echinochrome A (44), which can react to form peroxide in the presence of high calcium concentrations (45). Pigment cells are motile and exhibit a surveillance-like migratory behavior even in immunoquiescent conditions. However, in response to immune challenge (e.g., disturbance of gut bacteria or intracelomic bacterial injection), a subset of pigment cells increase motility enter the blastocoel and interact with other immune cells at sites of wounding or infection. These cells are morphologically and transcriptionally similar to the red spherule cells, which mediate wound healing and immune response in adults (25, 39).

During mid-blastula stage, a set of oral NSM cells are marked by expression of *gata1/2/3* and *scl* (25), transcription factors that are homologs of important vertebrate hematopoietic mediators. These cells undergo epithelial–mesenchymal transition later in gastrulation (about 10–15 h after the pigment cells) and enter the blastocoelar cavity where they differentiate into several cell types with immune activities. These include phagocytic cell types (a subset of *filopodial cells* and rarer, motile *ovoid cells* that appear upon acute immune challenge), highly motile *amoeboid cells* travel rapidly throughout the blastocoel, interacting with other immune cells and epithelia, and *globular cells*, a set of motile vesicular cell that are marked by expression of perforin/MPEG-like genes (17, 25). The phagocytic filopodial cells express the sea urchin-specific *Trf* genes in response to bacterial challenge, which parallels similar responses in adult phagocytic coelomocytes (34). Together, this assemblage of immune cell types dynamically interacts in the course of larval immune response.

Blastocoelar injection of labeled bacteria, fluorescent beads, or Zymosan (particles derived from yeast cell walls) into sea urchin larvae elicits immune cell migrations and phagocytosis (17, 23, 46, 47). In purple sea urchin larvae, the response varies according to the particle: *E. coli* K12 elicits a weak response whereas *Vibrio* species and Zymosan elicit much stronger responses (17). Injected *Vibrio diazotrophicus* cells agglutinate within minutes and are quickly engulfed by filopodial cells. Pigment cells and sometimes amoeboid and globular cells migrate and accumulate in regions of high bacterial concentration but are not phagocytic. Injection of Zymosan particles and *Vibrio* spp. cells sometimes elicit large, highly phagocytic cells (ovoid cells) that may derive from the syncytial filopodial cell network. The larval response to bacteria and other foreign particles involves layers of coordinated response among phagocytic and non-phagocytic immune cells and humoral factors.

## The Purple Sea Urchin Larva as a Model for Gut-Associated Immune Response

Four to five days after fertilization, the mouth opens and larvae begin to feed on algae and other planktonic organisms. Before this, the gut lumen is exposed to microbes through the open blastopore. Following the onset of feeding, however, the gut maintains significant contact with the microbial world. Immune cell activity at the gut epithelium and the complexity of immune gene expression in the epithelial cells highlight the importance of the gut in larval immunity. When larvae are cultured in freshly collected sea water (allowing them to feed on complex, natural food sources), pigment cells are commonly observed near the gut epithelium (rather than the ectoderm), indicating that the baseline state in wild populations is more immune activated than in quiescent laboratory animals.

An acute infection is induced by exposing larvae to high concentrations of the marine bacterium *V. diazotrophicus* [Figures 1C,D; Ref. (17)]. Within 6 h, the gut epithelium thickens and a subset of pigment cells, mainly those in the ectoderm nearest the midgut, migrate between the ectoderm and gut, making repeated filopodial contact with the midgut and hindgut epithelium. Amoeboid cells also increase contact with the gut epithelium and make dynamic contacts with pigment cells that can last for hours. While it is unclear what is communicated during this process, it highlights the complex, cell-type interactions involved in immune response in this morphologically simple organism. After about 20 h, bacteria appear within the blastocoel of most larvae where they are quickly phagocytosed by *Trf-*expressing filopodial cells. This response requires live bacteria and is reversed by removing bacteria from the seawater. Because the response is relatively synchronous, tens of thousands of larvae can be analyzed in parallel to assess global transcription changes even of rare transcripts.

## IL17 Cytokines Mediate the Larval Gut-Associated Inflammatory Response

Within 2 h of exposure to *V. diazotrophicus*, changes in gene expression are evident in peripheral pigment cells near the ectoderm (17). However, at this point in infection, bacteria are restricted to the gut lumen and are not observed in the blastocoel until much later (12–24 h post-exposure). This suggests the possibility that gut epithelial cells communicate the perturbed state in the gut lumen to the wider organism. To identify an early immune signal, an RNA-Seq assay was used to quantify system-wide transcript levels in larvae over a time-course of exposure to *V. diazotrophicus*. From these data, a small family of genes orthologous to vertebrate IL17 cytokines emerged as the most highly upregulated transcripts across the entire genome (29). The mammalian IL17 signaling molecules [IL17A–F (48)] are expressed in Th17 cells, and other lymphocytes, myeloid cell types, and barrier tissues (49–51), including gut epithelial cells (52–54). IL17 expression in epithelia, particularly IL17C in the gut, maintains barrier integrity and regulates microbiota composition (52–56).

**Figure 2:**
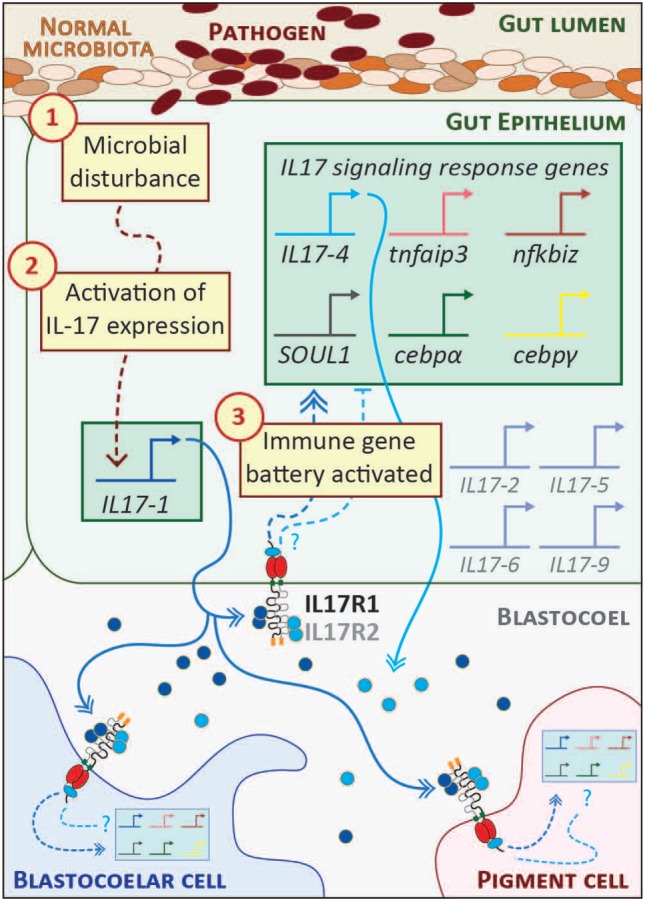
Interleukin 17 (IL17) signaling mediates the larval immune response. A hypothetical scheme of the signaling molecules and transcriptional events that occur during the initial phase of the larval gut-associated immune response is shown. The community of normal microbiota is shown within the gut lumen in shades of brown. The introduction of pathogenic bacteria (indicated in dark red) to the gut is sensed by receptors the gut epithelial cells as a microbial disturbance [indicated by step (1)]. A signaling cascade is initiated that results in the transcriptional upregulation of the *IL17-1* genes [step (2)]. This is evident within 2 h of seawater exposure to *Vibrio diazotrophicus*. IL17-1 protein (dark blue) is secreted, where it can interact with widely expressed IL17 receptors and affect gene expression in cells distributed across the organism. IL17-R1 and -R2 are shown here as heterodimers, although they may also homodimerize. Upon activation, these receptors initiate intracellular signaling pathways that result in the upregulation of an IL17-dependent gene battery [step (3); shown in the green box]. These genes were identified using *in vivo* perturbation of IL17-R1 signaling (29). Notably, this includes the *IL17-4* gene, which is always activated subsequent to *IL17-1*. This linkage may point to regulatory feedback between the two subtypes and, given the rapid attenuation of *IL17-1* transcripts, the IL17-4 protein (light blue) may serve as an inhibitory mechanism. Given the broad expression patterns of the IL17 receptors, it is likely that immune cells (blastocoelar cells are shown in blue; pigment cells, pink) contain cell-type specific regulatory circuitry that controls immune gene expression in response to IL17 signaling. Spliced messages from the other IL17 subtypes (gray) can be recovered from larvae, although the levels are very low. These may be activated under different immune challenge conditions.

The *S. purpuratus* genome contains 30 genes predicted to encode functional IL17 factors (and five pseudogenes) (23, 29). Ten subtypes (IL17-1–10) are differentially expressed in the sea urchin immune response. These genes are transcriptionally inactive in immunoquiescent animals and are absent from non-challenged *S. purpuratus* transcriptome data. In the larval response to *V. diazotrophicus*, genes within two subtypes are rapidly upregulated. The *IL17-1* genes (11 nearly identical genes) are activated within 2 h of exposure and then rapidly attenuated by 8–12 h. The single *IL17-4* gene is activated with a moderate delay relative to *IL17-1* and coincides with the upregulation of a battery of other immune genes. Both IL17 subtypes are expressed exclusively in the mid- and hindgut epithelium (29). Although some cells express only one subtype at any one time as assessed by *in situ* hybridization, these IL17 subfamilies are often co-expressed (Figure 2). The successive expression of these IL17 subtypes in the gut epithelium suggests the possibility of a feedback mechanism to regulate the response.

Genes within a third subfamily, *IL17-9*, are upregulated in adult sea urchin coelomocytes. Transcript quantification in coelomocytes collected from adult sea urchins challenged with either live *V. diazotrophicus* or sham injection controls indicates that challenged animals rapidly activated the *IL17-9* transcripts (peak expression 4–6 h post-infection). By contrast, the *IL17-9* genes were upregulated more slowly in sham-injected controls (12–24 h post-infection), which is consistent with a more attenuated expression of the *Trf* genes.

Vertebrate IL17 receptors are characterized by an intracellular signaling domain known as a SEF/interleukin-1 receptor (SEFIR) domain (57). Five widely expressed IL17 receptors (IL17RA–E) (58) dimerize to mediate signaling in mammals (58–60). The *S. purpuratus* genome contains two genes that encode SEFIR domains; domain architecture and phylogenetic analysis indicate that both are IL17 receptors (IL17-R1 and IL17-R2) (23, 29). Consistent with observations in vertebrates, the sea urchin IL17 receptors are expressed at low levels; whole mount *in situ* hybridization suggests a broad expression pattern with some enrichment in the gut.

The functional consequences of IL17 signaling were investigated within the context of the larval inflammatory response using morpholino antisense oligonucleotides to perturb IL17-R1 signaling (29). These reagents were microinjected into fertilized eggs, which were grown to larval stage and exposed to *V. diazotrophicus*. Candidate genes for expression analysis were chosen based on their expression patterns (a sharp upregulation just following *IL17-1* activation) or known transcriptional links in other systems. Larvae subjected to IL17-R1 perturbation exhibit decreased expression of immune genes in response to immune challenge relative to controls. In the absence of IL17-R1 signaling, immune-challenged larvae expressed reduced levels of *IL17-4*, which may point to regulatory or feedback interactions between the two IL17 subtypes. In addition, reduced expression was also evident for two IL17 target genes in vertebrates: *tumor necrosis factor α induced protein 3* (*tnfaip3*; also known as A20), which encodes a ubiquitin-editing enzyme that inhibits NF-κB activation (61), and *NF-κB inhibitor* ζ (*nfkbiz*), an IL17 target gene in vertebrates that also regulates NF-κB activity (62). Two IL17 associated transcription factors, *cebpα* and *cebpγ*, are exhibit reduced activation in the larval inflammatory response in the presence of perturbed of IL17R signaling (29). Finally, IL17 signaling regulates the expression of a gene known as *soul1* (29). This transcript encodes a protein that contains a heme-binding SOUL domain (PF04832). The functions of these evolutionarily widespread domains are not well understood in mammals (63). However, limiting iron availability is a known mechanism to suppress pathogen growth (64). The association between IL17 and SOUL1 may therefore represent an ancient regulatory connection yet to be identified in vertebrates. Together, these results indicate that highly regulated *IL17* expression in the sea urchin gut epithelium and signaling through IL17-R1 form a central axis of larval gut-associated immunity.

## Conclusion and Perspectives

The opening words in this review from Metchnikoff are now over 130 years old. Although Metchnikoff focused on cellular functions and we have long since moved to proteins and the genes encoding them, their relevance now holds renewed meaning. As we focus on genomes and the networks of regulatory interactions programmed therein, simple animal models offer novel strategies to investigate open problems in biology. These immune GRNs have evolved over hundreds of millions of years. Their highly complex and distributed nature requires that they be studied within intact organisms. The phylogenetic positions and experimental characteristics of well-chosen invertebrate models can be tailored to address specific questions. Here, we present the view that understanding these GRNs can shed light on how immune systems evolved on broad phylogenetic scales, a subject that remains poorly understood. The sea urchin larva is a morphologically simple model to experimentally characterize the system-wide GRNs that regulate immune cell development and immune response and is in an appropriate phylogenetic position to inform our understanding of vertebrate biology.

## Author Contributions

KB and JR wrote and edited the manuscript.

## Conflict of Interest Statement

The authors declare that the research was conducted in the absence of any commercial or financial relationships that could be construed as a potential conflict of interest.
